# The interRAI Acute Care instrument incorporated in an eHealth system for standardized and web-based geriatric assessment: strengths, weaknesses, opportunities and threats in the acute hospital setting

**DOI:** 10.1186/1471-2318-13-90

**Published:** 2013-09-05

**Authors:** Els Devriendt, Nathalie I H Wellens, Johan Flamaing, Anja Declercq, Philip Moons, Steven Boonen, Koen Milisen

**Affiliations:** 1 Department of Public Health and Primary Care, Health Services and Nursing Research, KU Leuven, Kapucijnenvoer 35, 4th floor, Leuven, 3000, Belgium; 2 Department of Internal Medicine, Division of Geriatric Medicine, University Hospitals Leuven, Leuven, Belgium; 3 Department of Clinical and Experimental Medicine, KU Leuven, Leuven, Belgium; 4 Lucas-Centre for Care Research and Consultancy, Policy Research Centre for Welfare, Public Health and the Family, Centre for Sociological Research, KU Leuven, Leuven, Belgium; 5 Leuven University Centre for Metabolic Bone Diseases, KU Leuven, Leuven, Belgium

**Keywords:** Aged, Comprehensive geriatric assessment, Hospital, InterRAI Acute Care, Software, SWOT-analysis

## Abstract

**Background:**

The interRAI Acute Care instrument is a multidimensional geriatric assessment system intended to determine a hospitalized older persons’ medical, psychosocial and functional capacity and needs. Its objective is to develop an overall plan for treatment and long-term follow-up based on a common set of standardized items that can be used in various care settings. A Belgian web-based software system (BelRAI-software) was developed to enable clinicians to interpret the output and to communicate the patients’ data across wards and care organizations. The purpose of the study is to evaluate the (dis)advantages of the implementation of the interRAI Acute Care instrument as a comprehensive geriatric assessment instrument in an acute hospital context.

**Methods:**

In a cross-sectional multicenter study on four geriatric wards in three acute hospitals, trained clinical staff (nurses, occupational therapists, social workers, and geriatricians) assessed 410 inpatients in routine clinical practice. The BelRAI-system was evaluated by focus groups, observations, and questionnaires. The Strengths, Weaknesses, Opportunities and Threats were mapped (SWOT-analysis) and validated by the participants.

**Results:**

The primary strengths of the BelRAI-system were a structured overview of the patients’ condition early after admission and the promotion of multidisciplinary assessment. Our study was a first attempt to transfer standardized data between home care organizations, nursing homes and hospitals and a way to centralize medical, allied health professionals and nursing data. With the BelRAI-software, privacy of data is guaranteed. Weaknesses are the time-consuming character of the process and the overlap with other assessment instruments or (electronic) registration forms. There is room for improving the user-friendliness and the efficiency of the software, which needs hospital-specific adaptations. Opportunities are a timely and systematic problem detection and continuity of care. An actual shortage of funding of personnel to coordinate the assessment process is the most important threat.

**Conclusion:**

The BelRAI-software allows standardized transmural information transfer and the centralization of medical, allied health professionals and nursing data. It is strictly secured and follows strict privacy regulations, allowing hospitals to optimize (transmural) communication and interaction. However, weaknesses and threats exist and must be tackled in order to promote large scale implementation.

## Background

Given the evolution of comprehensive geriatric assessment (CGA), three generations of CGA instruments are currently used in practice. First-generation CGA instruments use a collection of individually validated instruments that each focus on a single clinical domain of the patient (e.g. Mini Mental State Examination testing cognition [1], mini nutritional assessment evaluating nutritional status [2]) [3]. The assessment of a specific domain is usually triggered by the ‘impression’ of clinicians [4]. Second-generation geriatric assessment instruments include all geriatric domains, are setting-specific [5] and have been validated in each specific setting (e.g., MDS 2.0 [6]) [3]. While the first and the second generation of instruments allowed a systematic and standardized assessment of the patient, the items of the different instruments lacked the uniformity needed to transfer information across different settings (e.g. home care, long time care and acute care). Instruments of the third generation, such as the interRAI Suite, facilitate data transfer between healthcare settings, based on a common set of standardized items [5,7]. Supported by electronic standardized clinical data systems, assessment data can follow the patient across multiple care settings and optimize the coordination and quality of care. Although third generation CGA instruments exist, instruments of the first and second generation are still widely used.

The interRAI Suite consists of CGA instruments of the third generation, designed for a range of clinical services across multiple care settings [7]. One of these instruments is the interRAI Acute Care (interRAI AC) instrument, released in 2006 in order to identify the needs of older and disabled people admitted to acute hospitals. It is one of the most recent instruments of the interRAI portfolio [8] and, as a multidimensional CGA system, intends to determine a hospitalized older persons’ medical, psychosocial, and functional capacity and needs [9]. Its ultimate goal is to develop an overall plan for treatment and long-term follow-up based on a common set of standardized items that are used in various care settings.

A Belgian web-based software system, the BelRAI-software, was built to realize the assessment and transfer of uniform patient data across the home, residential and hospital settings. To the best of our knowledge, this software platform constitutes the first initiative to allow crossmural standardized data transfer for clinical purposes. The aim of this study was to provide an in-depth evaluation of the feasibility of the interRAI Acute Care instrument -integrated in the BelRAI web-based software system- in clinical practice in acute care hospitals.

## Methods

### Instrumentation

The interRAI AC instrument was previously translated and adapted to the Belgian (Flemish region) acute care hospital context [10]. Aspects of validity and reliability of the Belgian interRAI AC instrument have been reported before [8,11-14].

A Belgian web-based software system (BelRAI-software) was developed to provide a uniform web-based (online) registration of patient data and assessments from the interRAI instruments e.g. interRAI Acute Care (interRAI AC), interRAI Home Care (interRAI HC) and interRAI Long Time Care Facilities (interRAI LTCF). The BelRAI-software was developed in 2008 for home care organizations (interRAI HC) and nursing homes (interRAI LTCF). Only at a later stage, the interRAI AC instrument for hospitals was integrated into the system. The pilot project was the first to test the interRAI AC software in an acute care hospital setting. The interRAI AC software enables clinicians to map a geriatric patient in the hospital based on 98 different standardized clinical items over 12 domains. Four assessment periods (preadmission, admission, reassessment, and discharge, respectively) map the fluctuations of the patient’s status during the hospital stay. Once the assessment is completed, outcome measures (outcomes) can be calculated based on a composition of items across domains [9]. For each domain, clinical outcome measures are generated in the form of scales and clinical assessment protocols (CAPs) designed to support clinical decision making for frail older patients in the acute care setting. Scales represent the severity of illness or disability of the patient and the evolution of the illness over time. CAPs identify geriatric syndromes with the possibility to take preventive measures or to intervene. Benchmarking can be performed on individual patient data or on a group (ward or hospital) level. A health summary report is given after each assessment. The system also offers the opportunity to communicate about the patient’s health condition across wards and across care organizations (data transfer). A core set of items has been standardized across all instruments, enabling the uniformity of the assessment and the transfer of data. About 90-95% of the interRAI AC items are identical to those in the interRAI HC and LTCF. The latter instruments both contain substantially more items, up to 300. The items that are unique to the interRAI AC are for example being confined to bed for medical reasons, length of stay at emergency department, etc. Due to the uniform coding system, transfer of information to or from other participating organizations is possible for each older patient included in the BelRAI-system. All assessments of one patient are grouped and centrally stored in the BelRAI-system. All involved health professionals (within and outside the hospital) with permission to access the software, can consult this history of previous assessment data, on condition the patient has agreed that his or her file can be shared with others involved in his or her care. Both recent and older assessments are saved, and all items of each instrument are accessible. When consulting the record of a specific patient, an overview is given of all assessments labeled with date of the assessment, care setting, name of assessors and person responsible for the record. A previous assessment can be consulted in its entirety or the history of a specific item over different assessments can be checked. A history button on the screen allows the assessor to look at previous assessment dates, assessors’ names, type of instruments and scores. A health summary report of overall functioning and potential problem areas can be generated. This can be used as a transfer document.

The access to the BelRAI-system is limited. Care professionals get access with their electronic identity card, with which the Belgian E-health systems checks their identity and subsequently, their profession via authentic sources (e.g. an official list that identifies each profession involved). Consequently, a person only gets access to the data of patients if he has a current care relation with the patient and on the condition the patient gave informed consent. The data moreover is encrypted and stored in secured servers. Access to aggregated data, e.g. for research, is only possible with the approval of the privacy commission.

All privacy and legal requirements are fulfilled and strictly controlled by the Belgian e-health system (https://www.ehealth.fgov.be/nl/home).

**Figure 1:**
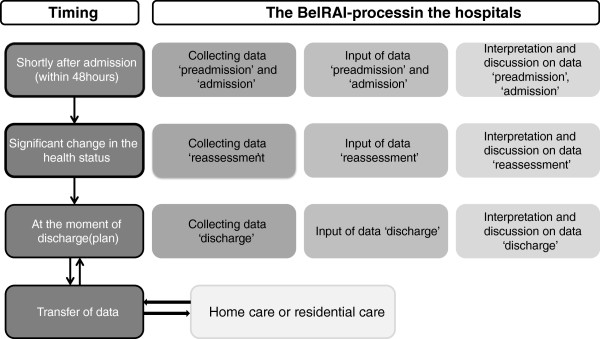
The BelRAI-process.

The ‘BelRAI-process’ in the hospital context consists of five steps: (1) collecting the data based on the interRAI Acute Care instrument (98 items), (2) input of these data in the BelRAI-software, (3) interpretation and discussion of outcomes (CAPs and Scales), (4) the use of these outcomes in team discussions, and (5) transfer of the data to other settings (Figure 1). As mentioned above, patients are assessed at multiple points in time during hospital stay (Figure 1).

### Procedure

A cross-sectional multicenter study was done. A pilot project during one year (from June 2010 until July 2011) was set up with homecare organizations (n = 14), nursing homes (n = 5) and hospitals (n = 3). In three acute care hospitals, on four geriatric wards, trained clinical staff of multiple disciplines [(head)nurses (n = 14), occupational therapists (n = 2), social workers (n = 4) and geriatricians (n = 9)] assessed 410 hospitalized older persons. The interRAI AC assessment was conducted in routine clinical practice.

Because of the complexity of the BelRAI-process, every caregiver had to pass through a learning process in order to manage the interRAI AC instrument and the BelRAI-software. Each participant was extensively trained during a 3-day course, including information on the interRAI instruments, the BelRAI-software, the security and privacy measures, and practical exercises on coding, patient cases and hands-on training. Participating hospitals were asked to gradually increase the number of BelRAI-assessments each month (e.g. 1st month: four assessments, 2nd to 4th month: six assessments each month, 5th month: eight assessments, …). Training and support during the project was organized at the request of the participating organizations.

With regard to technical and other issues, hospital staff was supported by a helpdesk with daily availability by telephone or by e-mail. Participants could consult a wiki-website specifically designed to improve the data quality [12], with a built-in electronic manual, providing information about the BelRAI-instruments, the assessment and the BelRAI-software. During the project, the three hospitals were contacted monthly to evaluate, support and adjust the BelRAI-implementation in the participating wards.

### Target population

Patients aged 75 years or older and verbally testable who were admitted to one of the participating acute geriatric units or with a geriatric profile according to a geriatric consultation team were included. Patients not speaking the local language, not verbally testable, transferred from another ward, or in very poor health condition (e.g., extreme pain, fatigue, dyspnea, medically unstable) were excluded.

### Ethics

The study was approved by the Medical Ethics Committee of the Leuven University Hospitals and all participating hospitals. All participating patients or their proxies provided written informed consent.

### Evaluation techniques

The balanced and detailed opinions of different participants with varying clinical backgrounds, all working in the hospital but playing a different role in the BelRAI-project (coordinator, assessor, …) were evaluated with the following three evaluation techniques: questionnaires, focus groups and interviews. Both the AC assessment and the BelRAI software were evaluated. Interactions with home care organizations and nursing homes were taken into account, based on data transfer from and to the hospital. The evaluation techniques, the healthcare workers who took part in the evaluation and the main topics are summarized in Table 1.

**Table 1:** Overview of the evaluation techniques

**Type**	**Healthcare workers taking part in the evaluation**	**Number of healthcare workers taking part per setting**	**Main topic**
**Questionnaire 1**	Assessors Healthcare workers	AC n= 16/20	Time investment
		HC n= 21/57	
		NH n= 60/109	
**Questionnaire 2**	Assessors Healthcare workers	AC n= 19/20	Evaluation of the BelRAI-process: demographic data, involvement of people in the BelRAI-process, own participation in the project, outcome measures, team discussion, transfer of data, evaluation of the BelRAI-software, helpdesk, comprehensive geriatric assessment, preconditions, barriers and levers and conclusions
		HC n= 44/57	
		NH n= 70/109	
**Focus group 1**	Local project coordinators	AC n= 3	Implementation, internal communication & financial implications
		HC n= 3	
		NH n= 5	
**Focus group 2**	Assessors Healthcare workers	AC n= 12	Evaluation of training & the progress of the BelRAI-process
		HC n= /	
		NH n= /	
**Focus group 3**	Assessors Healthcare workers	AC n= 6	Evaluation transfer of data
		HC n= 11	
		NH n= 5	
**Focus group 4**	Middle management	AC n= 3	Preconditions for implementation of BelRAI
		HC n= /	
		NH n= /	
**Focus group 5**	Assessors Healthcare workers	AC n= 5	Final evaluation of the project & the BelRAI-instrument
		HC n= /	
		NH n= /	
**Semi-structured interview**	Geriatrician	AC n= 6	Evaluation of the BelRAI-process & future implementation
		HC n= /	
		NH n= /	

*AC =* Acute care *= hospitals, HC =* Home care*, NH =* Nursing homes.

### SWOT analysis

In this study, a SWOT (Strengths, Weaknesses, Opportunities, Threats) analysis was used to summarize all results of the focus groups, interviews and questionnaires, which was constructed by the researchers reflecting the results of the participants’ opinions. It gives an overview of the feasibility of the interRAI AC instrument and its BelRAI-software in routine clinical hospital practice. The accuracy and correctness of the SWOT analysis was validated by the participating wards by asking the participants to provide feedback on the strengths, weaknesses, opportunities and threats of the BelRAI-process. Separate SWOT analyses were generated for the interRAI AC instrument and for the BelRAI-software, respectively.

### Analysis

For each focus group and interview, a topic list was prepared and additional questions were asked until saturation was reached. The focus groups were all transcribed verbatim and were coded independently by two researchers (ED and NW), themes were identified and the codes were assigned to the themes. These qualitative analyses were done using QRS NVIVO 8.

## Results

### Sample characteristics

The response rates for the first and second questionnaire in the hospital setting were 80% (n = 16/20) and 95% (n = 19/20), respectively. Most respondents were women 90% (n = 17). The average age of the healthcare workers was 39 years (range = 24-56, SD = 12). Six health professionals (30%) were employed part-time. Professional experience in care for older persons ranged between 2 and 5 years for six health professionals, between 5 and 10 years for three health professionals, and more than 10 years for nine health professionals.

### Completing the BelRAI-assessment

In the three participating hospitals 194, 173 and 43 older persons were assessed, respectively. A complete assessment (preadmission, admission, reassessment and discharge) according to the respondents was not always possible, due to the workload and the fast turnover of patients. All above mentioned older persons received a premorbid and admission assessment, a reassessment was done in only two cases, and 17 older persons did not receive a discharge assessment.

**Figure 2:**
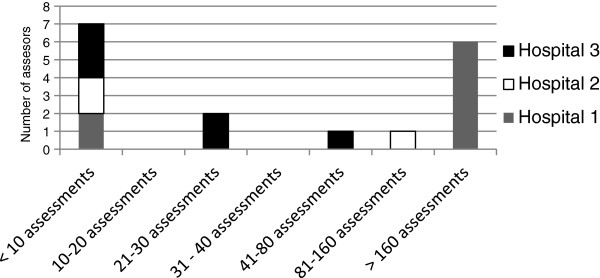
Distribution of the number of assessors according to the number of completed interRAI AC assessments in the BelRAI-software throughout the project.

The experience with the BelRAI-assessment acquired by the healthcare workers during the project time varied widely. The number of interRAI AC assessments in the BelRAI-software varied from less than 10 assessments per caregiver (n = 7) up to more than 160 assessments (n = 6) (Figure 2).

### Transfer of patient data

The BelRAI-assessments of 159 patients (39%) were exchanged with home care organizations (n = 127) and nursing homes (n = 32). The participating hospitals received 35 assessments coming from home care (n = 4) or residential care (n = 31). This information was evaluated as useful by six health professionals, while nine other health professionals stated they did not receive a sufficient number of assessments in order to be able to evaluate the quality of the received information. The health summary (n = 7), the CAPs (n = 8) and the items (n = 4) were consulted for collecting information about the patient coming in from another setting. Discussions in focus groups revealed that receiving an assessment from another setting made it possible to get a timely first insight in the overall health status of the patient and to detect problems at an early stage (e.g. early after admission in the hospital). Adaptations to the BelRAI-software could optimize this step in the BelRAI-process by making it faster and more efficient, e.g. by installing an automatic mailing system to fasten the transfer of data.

### SWOT analysis

**Figure 3:**
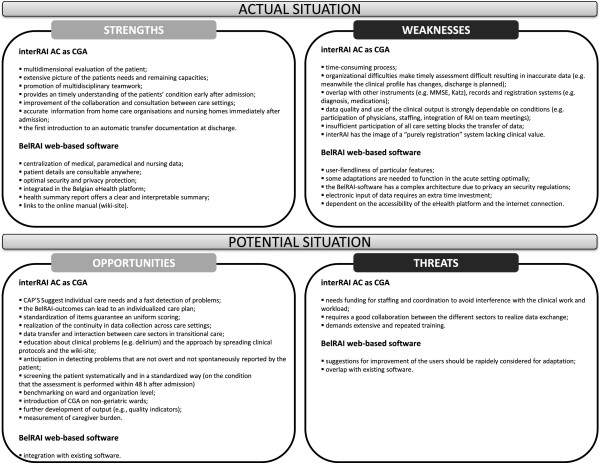
SWOT analysis.

The SWOT analysis for the BelRAI-pilot implementation was reported and summarized separately for the interRAI AC instrument and the BelRAI-software in Figure 3. In the result section below, the results were reported according to their importance for practice.

### Strengths

Primary strengths of the use of the BelRAI-system were (1) an understanding of the patients’ condition early after admission and (2) the promotion of multidisciplinary teamwork. In addition (3), for participating hospitals, the BelRAI-system was a first introduction to standardized data transfer with other care settings (e.g. home care and nursing home setting) and (4) a way to centralize medical, allied health professional, and nursing data. Finally, (5) the secure nature of the BelRAI-software was considered a particular strength as well, because of its strict privacy regulations and its integration in the Belgian eHealth Platform.

Preadmission and admission assessments were conducted between 24 and 48 hours after admission, as mandated in the interRAI guidelines. Health professionals got a global picture of the patient and could adapt the care planning early in the admission period (e.g. allowing early detection of a patient walking independently with an assistive device at home). All domains concerning the patient were mapped out, not only a selection of domains suspected to be problematic, allowing, for example, a patient with cognitive impairment before admission to be examined immediately and more extensively during the hospital stay.

All participating wards worked in a multidisciplinary way to complete the BelRAI-assessment, but the practical organization differed. Two hospitals collected data in a multidisciplinary way; in the other hospital the data collection was performed by one person who consulted other disciplines. Depending on the ward, the input of data was either done by one or by more health professionals. Data collection by multiple disciplines was considered to be more positively because of a higher accuracy of the data.

Because a systematic standardized transfer of data with other settings was new to all participating hospitals (some hospitals did exchange some data before but only based on open entry fields), our study was the first opportunity to test a standardized way of data transfer between different settings. The completed data transfers were considered to be clinically useful and time saving by the hospital care team. Overall, data transfer using the BelRAI-software was considered to improve collaboration between different organizations significantly.

Before using the BelRAI-software, most hospitals stored data from the different health professionals in a fragmented fashion, while some used electronic records, others worked with paper files. The introduction of the BelRAI-software resulted in centralization of data input from nurses, physicians and allied health professionals into a common electronic patient record. The centralization of the data made it possible for health professionals to consult relevant data entered by other health professionals. Through the internet, authorized healthcare workers in other organizations outside the hospitals had access to relevant patient data as well.

It was of great importance that the BelRAI-software was strictly secured and strict privacy regulations were followed.

### Weaknesses

The most important weaknesses were considered to be (1) the time consuming character of the process, (2) the limited collaboration of physicians and (3) the overlap with other assessment instruments or (electronic) registration forms. Room for improvement was also identified for (4) the user friendliness and (5) the efficiency of the BelRAI web-based software system, with an additional need for (6) hospital-specific modifications to the software.

Because an acute care hospital is characterized by a short hospital stay, high turnover and consequently a high workload, the interRAI instruments were designed to collect 97% of the necessary data through observations during usual care. During the focus groups, health professionals confirmed that the instrument was mostly used as a systematic checklist in routine care, with the remaining 3% of the items easily obtained from the patient or from the available records. But time is needed to train health professionals in using the instrument and the software. This learning phase requires a substantial investment of time. Going through security procedures, the input of medication and diagnosis are time consuming and improvements in user-friendliness and efficiency were considered important.

Because interRAI prescribes that patient status should be assessed within 24 hours after admission, user-friendliness and efficiency are even more critical. In our project, organizational obstacles were often found to interfere with early assessment at the time of admission (e.g. the absence of assessors, need to give priority to clinical examinations, …). Overall, BelRAI-outcomes typically only became available during a later stage of the hospitalization when the acute episode was already over and (some of) BelRAI-outcomes had already become less relevant.

Although active participation of physicians is important in the BelRAI-process, involvement of physicians in our pilot project was low or even non-existent. In most cases, nurses completed all medical data based on the medical file or by consulting the attending physician, questioning the accuracy of some of the medical information. As the physician leads and supervises the clinical process, his or her support is crucial.

Most of the hospitals were already familiar with some kind of geriatric assessment, but often not in a systematic, standardized and consistent way and not immediately upon admission. Different assessment instruments of the first generation are used in order to evaluate specific domains, including cognitive evaluation using the MMSE [1] and physical functioning using the Katz-scale [15]. Even when they used the interRAI AC, they continued using these first generation instruments. This was perceived as double work and as a waste of time. Integration with different (electronic) systems was considered a possible solution. Stop using first generation instruments could be an alternative solution.

During this project the BelRAI-software for acute care was tested for the first time. All participants (n = 19) suggested changes to the BelRAI-software. Participants emphasized that the BelRAI-process needs to be carried out fast, safe, without technical problems and with as few steps as possible, given the large workload in the acute setting.

### Opportunities

The greatest opportunities for the BelRAI-instrument we identified were a timely and systematic detection of (early) problems (1), the early development of a care plan (2) and its contribution to continuity of care (transfer of data) (3).

Screening a patient systematically and in a standardized way makes it possible to detect problems that are not obvious and not reported spontaneously by the patient.

Assessment immediately after admission allows timely detection of problems and the development or adjustment of an individualized care plan. For instance, if a patient is admitted because of a fall and the interRAI assessment indicates cognitive problems, further assessment of cognitive performance can be done. However, during this project, BelRAI-health professionals seldom reviewed and analyzed the outcomes. A quarter (n = 5) of the participants never consulted the CAPs, 30% (n = 6) rarely consulted the CAPs, 20% (n = 4) did this occasionally and only 10% (n = 2) consulted the CAPs for most of the patients. Most of the health professionals, consulting the CAPs occasionally or regularly, considered the CAPs just to confirm risk factors. Only 35% (n = 7) discussed the CAPs once during a team meeting, while more than 50% (n = 10) never did. Scales were less frequently used, 15% (n = 3) consulted the scales occasionally or rarely and 40% (n = 8) never did. Again, for many health professionals, the scales simply confirmed their clinical feelings. Scales were never discussed during team meetings. Seventy-five percent of the health professionals never consulted the individual or ward benchmarking data and none of the participants introduced a systematic use of the BelRAI-outcomes during the weekly team meetings. Time pressure (n = 11) was the major reason why outcomes were not discussed at team meetings. BelRAI-output was rarely used during this study, suggesting that efforts are needed to coach geriatric teams how to integrate the BelRAI-output in daily practice and care planning. BelRAI could support this by further developing the output delivered by the system.

A major opportunity provided by BelRAI is transfer of patient data across settings or, within a hospital setting, across different wards, in particular because of standardization of items between different settings e.g. the scoring system to evaluate the patient’s physical functioning becomes identical in home care organizations, residential care organizations and the hospital.

### Threats

Although health professionals could base the assessment on clinical observations, a lack of funding to allow dedicated staff to coordinate the assessment process as well as the shortage of assessment personnel for the scoring of the items were seen as the most important threats. In the initial phase, another burden is a roll-out of the system, which is time consuming as well. An investment in time for training, for a learning phase and for the change in organization of daily work is necessary. In addition, the complexity of the BelRAI-process requires continuing training and permanent education. Health professionals underline the importance of ‘practical’ training and exercises with the instrument and the software. Theoretical background about the development of the system was to a large group of participants considered as less important. Two to three days of training in small groups was seen as an appropriate duration.

To be useful across different settings, the BelRAI-process is strongly dependent on collaboration within and between the organization(s) (e.g. collaboration of physicians). Lack of collaboration caused problems with the efficiency of the BelRAI-process and the transfer of data. In this context, nationwide implementation across all geriatric care settings requires high-level support from policy makers.

## Discussion

The interRAI suite is a 3rd generation standardized CGA instrument designed to support holistic care planning and data transfer across settings [7,16]. To date, no other system exists that made data transfer across settings possible. In this regard, the BelRAI-software is the first attempt to standardize data transfer between hospital, home care and residential care. Because a nationwide implementation is considered, our study was intended to provide an extensive evaluation of a pilot-implementation.

A SWOT-analysis in Belgian acute care hospitals identified a set of barriers for the implementation of the BelRAI-process, but also helped to reveal multiple strengths and opportunities. Solutions are at hand for the perceived weaknesses and threats, which must be taken care of before an implementation on a larger scale can be considered.

Based on our SWOT-analysis, we first of all identified the need for dedicated funding of staff but also for the development and maintenance of software, hardware and security devices. Secondly, collaboration within the organization and between different organizations in different settings is needed. A multidisciplinary and cross-setting approach is essential to develop a system of continuous data transfer. Thirdly, the software developed for BelRAI must be adapted to the hospital context and should become more user-friendly. An improvement of the existing output (e.g. CAPs and scales) in the web-based software would enhance the understanding of the patient condition. A fourth precondition is the need for continued education and training, both theoretical and practical. A last precondition deals with the integration of the BelRAI-software with other software or discontinuation of older applications in order to prevent double encoding and to reduce extra time investment.

The most important barriers identified during the project were the high turnover of patients the heavy workload in the acute setting, with a shortage of staff, insufficient knowledge of the instrument and the software and a lack of insight in the situation of the client before hospitalization when no previous BelRAI-assessment was available. Lack of interest from other team members was another barrier for the BelRAI-implementation.

Transfer of data over different settings was hampered by a lack of collaboration between different care partners. Because we only included a limited selection of hospitals, home care organizations and nursing homes in this pilot project, our findings should be interpreted with caution and future research on a larger scale is needed to confirm our findings. Also, we specifically tested the BelRAI-process in geriatric services in the hospital. Future studies should evaluate the assessment process for geriatric patients on non-geriatric wards. This will require appropriate screening strategies to identify the older patients at risk on non-geriatric wards who would benefit most from the BelRAI-process.

This study has also limitations. Although the SWOT-analysis are recognized a useful tool to document the organization of health services and to develop action plans [17], little research is available on how to use the data in daily practice. Because a SWOT-analysis is an approach that is more intuitive and judgmental rather than mechanistic or measurable [17], the analysis can be a good starting point and helps identifying and prioritizing the information to guide choices [17]. However, we do acknowledge that the SWOT approach is a less powerful technique to evaluate the feasibility of the BelRAI-assessment method and that future studies will need to include cost-effectiveness analyses.

A second limitation is the lack of data on the sample size, sample characteristics and patients not consenting or dropping out from the study. The focus of this research project was on the evaluation of the implementation process but not on the representativeness of the sample.

Third, technical challenges to deal with incomplete assessments, inconsistencies and invalid codes were not addressed in the current study as these aspects of validity based on test content were extensively evaluated in a previous study [8]. These problems were tackled by adjusting the BelRAI-software aiming to improve the quality of data.

InterRAI AC as an electronic web-based software system can give hospitals the possibility to evaluate patients systematically, in a standardized way, across all domains, centralizing medical, allied health professionals and nursing data, avoiding duplicated data and exchanging data with other settings. Software could be an innovative contribution to the implementation of CGA instruments.

Before a nationwide implementation of the BelRAI-instrument can be considered, policy decisions will be required to support significant improvements and investments.

## Conclusion

The BelRAI-software is the first attempt to standardize transmural transfer of information and centralize medical, allied health professionals and nursing data, based on a secure system. Any implementation, however, will require improvements in user-friendliness and efficiency, and investments in staffing, training and education.

## Competing interests

The authors declare that they have no competing interests.

## Authors’ contributions

ED was responsible for the study concept and design, acquisition of data, analysis and interpretation of data, and drafting the manuscript. NW participated in the study concept and design, the acquisition of data, the analysis and interpretation of data, and drafting the manuscript. JF participated in the study concept and design, the acquisition of data, and the analysis and interpretation of data. AD participated in the study concept and design, and in the analysis and interpretation of data. PM participated in the study concept and design, the acquisition of data, and the analysis and interpretation of data. SB participated in the study concept and design, and in the analysis and interpretation of data. KM was responsible for the study concept and design, acquisition of data, analysis and interpretation of data and drafting the manuscript. All authors read, revised and approved the final manuscript. Supervision was done by KM.

## Pre-publication history

The pre-publication history for this paper can be accessed here:

http://www.biomedcentral.com/1471-2318/13/90/prepub
